# LRP2 and DOCK8 Are Potential Antigens for mRNA Vaccine Development in Immunologically ‘Cold’ KIRC Tumours

**DOI:** 10.3390/vaccines11020396

**Published:** 2023-02-09

**Authors:** Shichao Zhang, Kaide Xia, Yue Chang, Yimei Wei, Yu Xiong, Fuzhou Tang, Jian Peng, Yan Ouyang

**Affiliations:** 1 Key Laboratory of Infectious Immune and Antibody Engineering of Guizhou Province, Engineering Research Center of Cellular Immunotherapy of Guizhou Province, School of Biology and Engineering/School of Basic Medical Sciences, Guizhou Medical University, Guiyang 550025, China; 2 Clinical College of Maternal and Child Health Care, Guizhou Medical University, Guiyang 550025, China; 3 Immune Cells and Antibody Engineering Research Center of Guizhou Province, Key Laboratory of Biology and Medical Engineering, Guizhou Medical University, Guiyang 550025, China; 4 MOE Key Laboratory of Cell Activities and Stress Adaptations, School of Life Sciences, Lanzhou University, Lanzhou 730000, China

**Keywords:** mRNA vaccine, kidney renal clear cell carcinoma, tumour antigens, immune subtypes, immune landscape

## Abstract

The administration of mRNA-based tumour vaccines is considered a promising strategy in tumour immunotherapy, although its application against kidney renal clear cell carcinoma (KIRC) is still at its infancy stage. The purpose of this study was to identify potential antigens and to further select suitable patients for vaccination. Gene expression data and clinical information were retrieved from Gene Expression Omnibus (GEO) and The Cancer Genome Atlas (TCGA) databases. GEPIA2 was used to evaluate the prognostic value of selected antigens. The relationship of antigens presenting cell infiltration with antigen expression was evaluated by TIMER, and immune subtypes were determined using unsupervised cluster analysis. Tumour antigens LRP2 and DOCK8, which are associated with prognosis and tumour-infiltrating antigen-presenting cells, were identified in KIRC. A total of six immune subtypes were identified, and patients with immune subtype 1–4 (IS1–4) tumours had an immune ‘cold’ phenotype, a higher tumour mutation burden, and poor survival. Moreover, these immune subtypes showed significant differences in the expression of immune checkpoint and immunogenic cell death modulators. Finally, the immune landscape of KIRC revealed the immune-related cell components in individual patients. This study suggests that LRP2 and DOCK8 are potential KIRC antigens in the development of mRNA vaccines, and patients with immune subtypes IS1–4 are suitable for vaccination.

## 1. Introduction

Kidney renal clear cell carcinoma (KIRC) is a common renal malignant tumour, with an increasing incidence, which accounts for about 70% of the total number of renal cancer diagnoses [1]. Due to the asymptomatic early phase of KIRC, most cases are diagnosed at advanced stages. Late-stage KIRC is characterised by the triad of renal cancer, namely haematuria, low back pain, and an abdominal mass [2,3]. Although surgical resection can remove early tumours, about one-third of patients with cancer metastasis find it difficult to achieve the desired therapeutic effect [4]. At present, the therapeutic effect of chemotherapy and radiotherapy on advanced KIRC is not ideal [5]. Patients, especially those in advanced stages, are prone to poor prognosis and high mortality, which brings new challenges to early clinical discovery and treatment [5].

Currently, immunotherapies based on immune checkpoint inhibitors (ICIs) have been successful in combating some malignant tumours, but only a few KIRC patients benefit from ICIs therapy. After targeting the immune checkpoint proteins, RNA vaccines are considered another effective immunotherapy tool. Recent studies have shown that mRNA vaccines represent a relatively new class of vaccines that show great promise in situations in which traditional vaccine platforms may not be able to induce protective immune responses, and the use of mRNA vaccines could be a hotspot in cancer immunotherapy [6,7]. In fact, until a decade ago, there were few attempts at mRNA-based therapies due to their extreme instability and an excessive inflammatory response. However, recent breakthroughs, including the addition of modified nucleosides, optimisation of coding sequences, and strict purification of in vitro transcription (IVT) mRNA by high-performance liquid chromatography (HPLC) to remove double-stranded RNA (dsRNA) contaminants, have changed this situation [7,8]. mRNA sequences can be easily modified as needed to produce any protein, which can greatly improve the production efficiency of vaccines and shorten the waiting period of vaccines for cancer patients. More importantly, the half-life of mRNA can be regulated by a delivery system or RNA sequence modification [9,10]. The self-adjuvant properties of mRNA can enhance its immunogenicity in vivo and induce a strong and sustained immune response. Thus, vaccines encoding tumour-specific antigens have been shown to promote anti-tumour immunity in preclinical and clinical models of a variety of tumours, including melanoma and pancreatic adenocarcinoma [11,12,13].

However, there is currently no effective mRNA vaccine for KIRC because it is challenging to isolate effective antigens from the thousands of mutant candidate genes in tumour cells to use against KIRC [14]. Moreover, only a small percentage of KIRC patients are likely to benefit from mRNA vaccines because of the high heterogeneity of tumour cells and their complex tumour immune microenvironment (TIME) [15,16,17,18]. Therefore, the tumour biological subtype stratification can be used to identify patients who are suitable for mRNA vaccines. Based on specific molecular patterns, previous KIRC classifications have focused on the intrinsic molecular profile of tumour cells, including gene amplification, copy number alterations, and signalling pathway dysregulation, with a few focusing on tumour-infiltrating immune cells [4,19,20]. From the point of view of the interaction between tumour cells and the body’s immune system, these traditional methods are not sufficient to screen candidate genes for mRNA vaccines. In contrast, the stratification of immune gene expression profiles in patients allows for the identification of patients suitable for mRNA vaccination [21,22].

The purpose of this study is to screen new KIRC antigens that can be used to develop mRNA vaccines and to select suitable patients for vaccination by mapping their immune profiles. Two candidate genes associated with survival and antigen-presenting cell (APC) infiltration were identified from the pool of overexpressed and mutated genes in KIRC. Six robust immune subtypes and eight functional modules of KIRC were identified by immune-associated gene clustering, with each corresponding to different clinical, molecular, and cellular characteristics, and these subtypes were validated in an independent cohort. Our findings define the complex TIME of each KIRC patient and provide a theoretical basis for the selection of mRNA vaccine targets and the screening of patients suitable for the mRNA vaccine.

## 2. Materials and Methods

### 2.1. Data Acquisition

The RNA sequencing data of 530 KIRC patients and their corresponding clinicopathological information were acquired from The Cancer Genome Atlas (TCGA, https://www.cancer.gov/tcga; discovery cohort). We also downloaded the RNA expression data of 265 cases and their corresponding clinicopathological information from the Gene Expression Omnibus (GEO, https://www.ncbi.nlm.nih.gov/geo, accessed on 1 May 2021; GSE73731; validation cohort). Clinical information is shown in the Supplementary Table S1. Based on the previous literature, a total of 2598 immune-related genes were collected, such as TGF-β family members, chemokines and chemokine reports, antigen processing and presentation, and TNF family members, as well as other immune-related genes.

### 2.2. Data Pre-Processing

Microarray expression data from GEO was pre-processed and aggregated to exclude microarray probes with invalid gene detection values. We identified 782 immune-cell-related genes from the 21,655 expressed genes. Among TCGA tumour samples, samples with incomplete clinical information and survival times less than 90 days were excluded. In addition, genes with a mean FPKM < 0.5 were also removed.

### 2.3. GEPIA Analysis

Gene Expression Profile Interaction Analysis (GEPIA, http://gepia2.cancer-pku.cn, accessed on 5 May 2021) [23] was utilised to identify differentially expressed genes (DEGs) and to analyse the prognostic value of screened antigens. The DEGs were gained on the basis of the q-value < 0.01 and absolute value of log2 fold-change > 2. A Kaplan–Meier method was adopted to assess OS and RFS in KIRC patients with a 50% (median) cut-off value, and log-rank tests were used to determine statistically significant differences between high and low expression groups (*p* < 0.05).

### 2.4. Genome Alteration Analysis

Somatic mutation and copy number alterations were analysed using the cBio Cancer Genomics Portal [24] (cBioPortal, http://www.cbioportal.org, accessed on 7 May 2021), and *p* < 0.05 indicated a statistically difference.

### 2.5. Detection of Tumour Antigen Expression

Tumour specimens from 19 patients with KIRC from the affiliated cancer hospital of Guizhou Medical University were identified. The study was approved by the Ethics Review Committee of Guizhou Medical University (protocol code 2022105 and 1 March 2022) and received written informed consent from the patients in accordance with the Declaration of Helsinki. RNA was extracted from tumour tissue and normal tissue of each patient, then reverse transcription was performed, and corresponding primers were used for fluorescence quantitative PCR detection. DOCK8-F: ACAAGACGCTTCCGAAACAG, DOCK8-R: CAAAGTCCTCGGCACTCA. LRP2-F: GCCCTTTCGCTGTCCTAGTT, LRP2-R: GGGCTCTTGAACACACTCGT.

### 2.6. Immune Cell Infiltration Analysis

We used the Tumour Immune Estimation Resource [25] (TIMER, https://cistrome.shinyapps.io/timer/, accessed on 25 May 2021) to evaluate the association of infiltration abundance of immune cells with KIRC-associated genes. *p* < 0.05 indicated a statistically difference.

### 2.7. Identification and Validation of Immune Subtypes

To identify the different gene modules and corresponding immune subtypes, cluster analysis was conducted by constructing a consistency matrix based on the expression patterns of 2598 immune-related genes. Briefly, the 1-Pearson correlation coefficient with 500 bootstraps (each bootstrap with 80% item resampling) was first used to determine the distance metric. A range of K from 2–10 was then selected to identify the immune subtypes and was verified by the GEO cohort. In addition, the consistency of immune subtypes in the discovery and validation cohorts was evaluated by calculating the Pearson correlation and intra-group proportion.

### 2.8. Immune-Associated Signaling Pathways

The GO enrichment analysis (biological processes; BP) was performed using the ‘clusterProfiler’ package to explore the pathways of immune-associated molecular and cellular features [26]. We analysed the relationships of 56 immune-associated molecular features with immune subtypes and evaluated the composition of immune cells in tumour tissues applying the CIBERSORT algorithm [27].

### 2.9. Gene Co-Expression Network Analysis

The immune-related gene co-expression modules were constructed based on the ‘blockwiseModules’ function of the WGCNA package in R. An immune enrichment score was calculated for each sample by single-sample GSEA (SSGSEA) to measure the coordinated up- or down-regulation of genes in the sample.

### 2.10. Establishment of the Immune Landscape

Graph learning-based dimensionality reduction analysis was performed using the dimensionality reduction function of the ‘Monocle’ package with Gaussian distribution to visualise the distribution of immune subtypes among individual patients, with 2 as the maximum number of components. The immune landscape was achieved using functional map cell trajectories of immune subtypes, which were characterised by different colours.

## 3. Results

### 3.1. Identification of Potential KIRC Candidate Antigens

**Figure 1:**
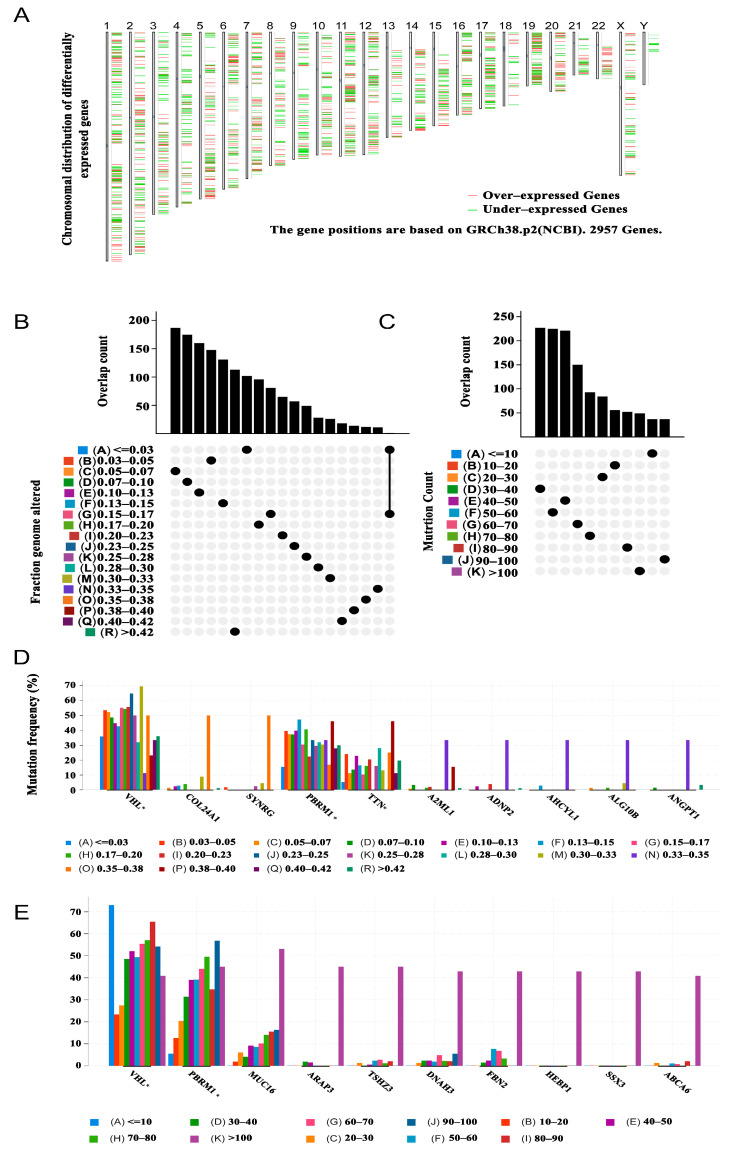
Potential tumour antigens of KIRC were identified from the database. (**A**) Chromosomal distribution of potential tumour-associated antigens in KIRC, with upregulation and downregulation indicated in red and green, respectively. (**B**) Summary of the distribution of mutant genes in the fraction-genome-altered group. (**C**) Summary of the distribution of mutant genes in the mutation-counting group. The highest frequency genes in altered genome fraction groups (**D**) and mutation count groups. (**E**) Kidney renal clear cell carcinoma (KIRC).

The workflow of this study is presented in Figure S1. To identify the potent antigens of KIRC from many candidates, 1633 overexpressed genes encoding tumour-associated antigens were first determined (Figure 1A). After analysis of the altered genome fraction and the number of mutations in a single sample, 9843 mutated genes encoding tumour-specific antigens were screened (Figure 1B,C). Mutational analysis showed that von Hippel–Lindau (VHL) and BRG1-associated factor 180 (PBRM1) were the most frequently mutated genes, both in terms of the altered genome fraction (Figure 1D) and mutation counts (Figure E). Most notably, the top 10 candidates with altered genome fractions included titin (TTN), collagen type XXIV alpha 1 chain (COL24A1), synergin gamma (SYNRG), alpha-2-macroglobulin like 1 (A2ML1), ADNP homeobox 2 (ADNP2), adenosylhomocysteinase like 1 (AHCYL1), ALG10 alpha-1,2-glucosyltransferase B (ALG10B), and angiopoietin 1 (ANGPT1; Figure 1D). In addition, in mucin 16 (MUC16), ArfGAP with a RhoGAP domain, ankyrin repeat and PH domain 3 (ARAP3), tea shirt zinc finger homeobox 3 (TSHZ3), dynein axonemal heavy chain 3 (DNAH3), fibrillin 2 (FBN2), haem-binding protein 1 (HEBP1), SSX family member 3 (SSX3), and ATP-binding cassette subfamily A member 6 (ABCA6), high mutation counts were observed (Figure 1E). Overall, 684 tumour-specific genes that were overexpressed and mutated with high frequency were identified.

### 3.2. Identification of KIRC Candidate Antigens Associated with Patient Prognosis and Antigen-Presenting Cells

**Figure 2:**
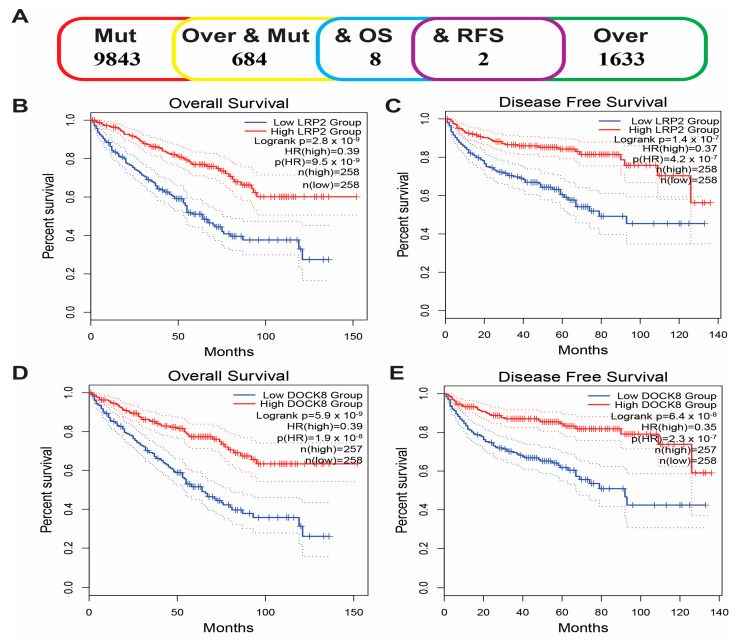
Identification of tumour antigens associated with KIRC prognosis. (**A**) Potential tumour antigens with overexpression and mutation are significantly associated with OS and RFS (2 candidate antigens). Kaplan–Meier curves showed that LRP2 expression was related to OS (**B**) and RFS (**C**) in KIRC patients. The Kaplan–Meier curve showed that the DOCK8 expression level was related to OS (**D**) and RFS (**E**) of KIRC patients. Kidney renal clear cell carcinoma (KIRC); Overall survival (OS); Relapse-free survival (RFS).

**Figure 3:**
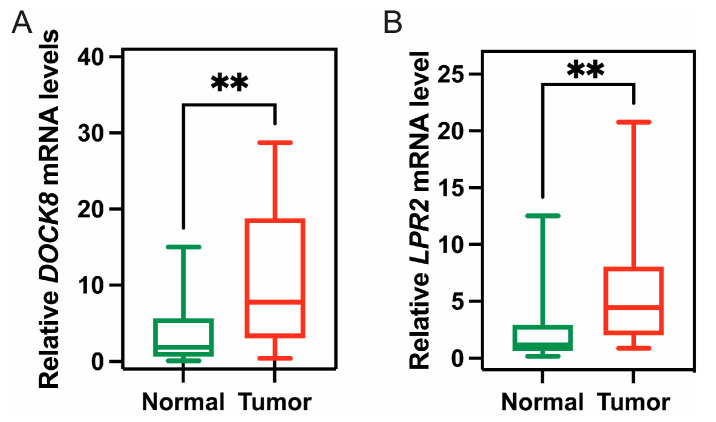
The expression of tumour antigen in clinical tumour patients. The expression of DOCK8 (**A**) and LRP2 (**B**) in tumour and normal tissues of 19 KIRC patients; (** *p* < 0.01).

**Figure 4:**
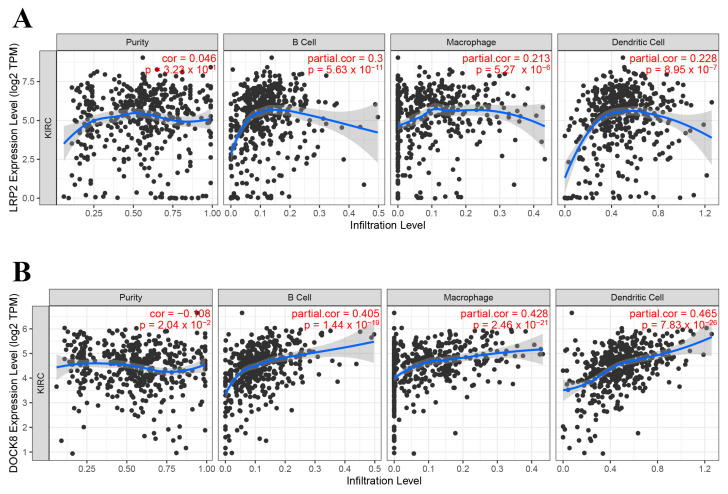
Identification of APC-associated tumour antigens. Correlation of LRP2 (**A**) and DOCK8 (**B**) expression levels in KIRC tumours with B cell, macrophage, and dendritic cell infiltration. Antigen-presenting cells (APC); Kidney renal clear cell carcinoma (KIRC).

From the above genes, tumour genes related to patient prognosis were selected as potential antigens for developing mRNA vaccines. Eight genes were closely related to OS in KIRC patients, and two of them were significantly related to RFS (Figure 2A). As shown in Figure 2B,C, patients with LDL-receptor-related protein 2 (LRP2) overexpression in tumour tissues had significantly longer survival than patients with low LRP2 expression. Similarly, high expression of the dedicator of cytokinesis 8 (DOCK8) was also associated with better prognosis (Figure 2D,E). Of note, we further detected the gene expression levels of tumour antigens in tumour and normal tissues of 19 KIRC patients and found that the expression levels of DOCK8 and LRP2 in tumour tissues were significantly higher than normal tissues (Figure 3A,B). In summary, two candidate genes were identified as having fundamental roles in the occurrence and progression of KIRC. In addition, the expression levels of LRP2 and DOCK8 were positively correlated with the abundance of tumour-infiltrating immune cells, including B cells, macrophages, and DCs (Figure 4A,B). These results suggest that the screened tumour antigens may be directly processed by APCs and then presented to T cells, which then interact with B cells to trigger an adaptive immune response. Therefore, these antigens are promising for development as mRNA vaccines against KIRC.

### 3.3. Identification of Potential Immune Subtypes of KIRC

**Figure 5:**
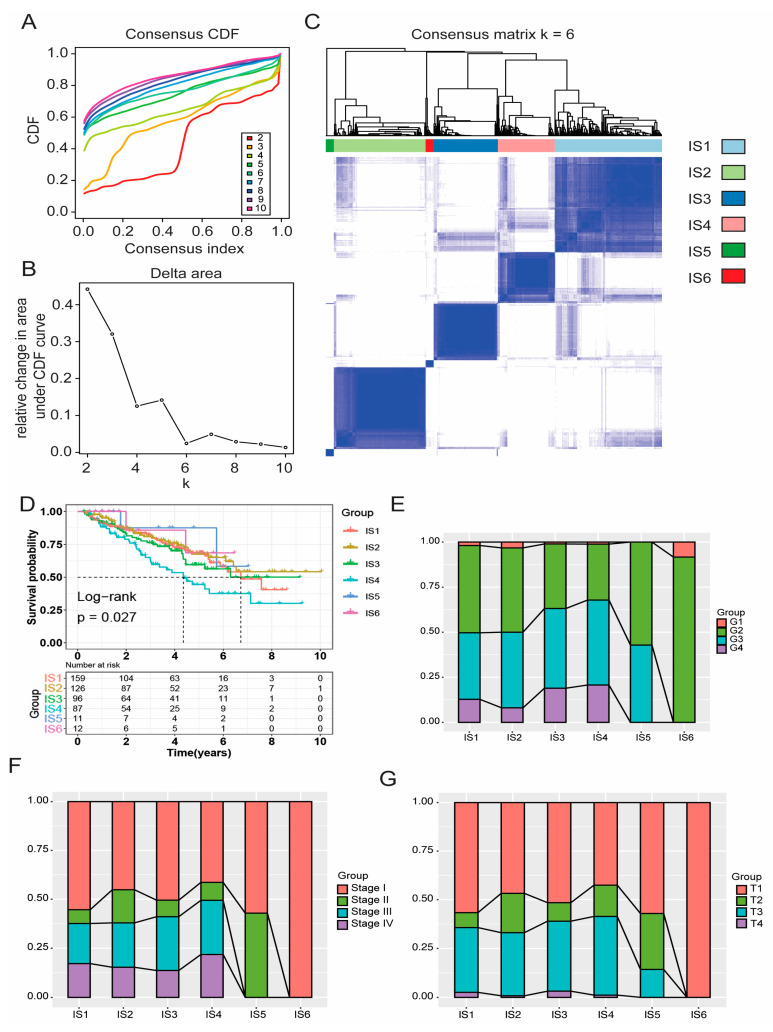
Identification of immune subtypes of KIRC. Cumulative distribution function curve (**A**) and triangular area (**B**) of immune-related genes in the TCGA cohort. (**C**) Sample cluster heat map of immune-related genes in the TCGA cohort. (**D**) Kaplan–Meier curve showing OS of KIRC immune subtypes in the TCGA cohort. In the TCGA cohort, the proportion of IS1–6 distribution in KIRC grade (**E**), stage (**F**), and T-stage (**G**). Kidney renal clear cell carcinoma (KIRC); The Cancer Genome Atlas (TCGA); Overall survival (OS); Immune subtype (IS).

Due to the large differences in tumour immune status and immune microenvironment, patients with KIRC have different immune responses. Therefore, patient immunophenotyping can help determine which subgroups are suitable for vaccination. We first collected 2598 immune-related genes on the basis of the previous literature. Then, on the basis of the expression profiles of these immune-related in 530 KIRC tumours from the TCGA database, immune subtypes were identified using a consensus-clustering method. According to their cumulative distribution function and functional delta area, immune-related genes showed stable clusters when k = 6 (Figure 5A,B), and six immune subtypes were obtained and defined as IS1–IS6 (Figure 5C). IS5 and IS6 were related to prolonged survival, whereas IS4 and IS2 had the worst prognosis (Figure 5D). The distribution of immune subtypes in different tumour stages and grades showed that IS5 and IS6 were significantly associated with lower tumour grades (Figure 5E), earlier clinical stage (Figure 5F), and T-stage (Figure 5G). In conclusion, tumour immunotyping can be used to predict the prognosis of patients with KIRC, and its prediction accuracy, which was consistent across cohorts, was better than conventional tumour grading and staging.

### 3.4. Correlation of Immune Subtypes with Mutational Status

**Figure 6:**
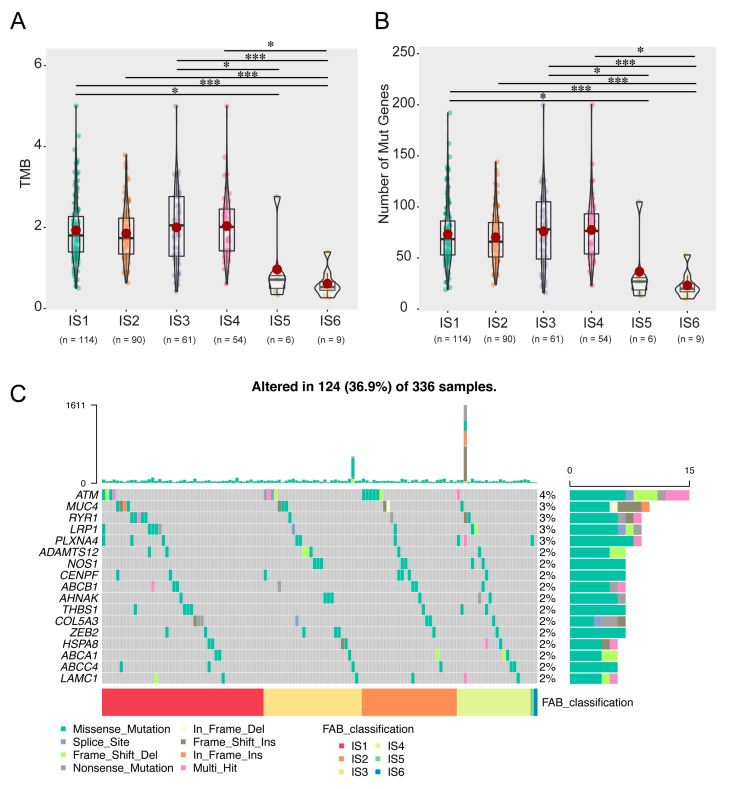
Correlation of immune subtypes with TMB and mutation. The TMB (**A**) and mutation numbers (**B**) in IS1–6 tumours. (**C**) Proportion of 17 highly mutated genes in KIRC immune subtypes. (* *p* < 0.05, *** *p* < 0.001). Tumor mutation burden (TMB); Immune subtype (IS); Kidney renal clear cell carcinoma (KIRC).

Previous studies have shown that higher tumour mutation burden (TMB) and somatic mutation rate are closely associated with immunotherapy efficacy and an enhanced immune response [28]. Therefore, the TMB and mutations of each patient in all immune subtypes were calculated using TCGA’s MuttecT2 mutation dataset. As shown in Figure 6A, compared with IS1–4, the TMB and mutant gene numbers of IS5 and IS6 were significantly reduced (Figure 6B). Furthermore, 17 genes, including ATM serine/threonine kinase (ATM), were more frequently mutated in each subtype (Figure 6C). These results suggest that patients with immune subtypes IS1–4 may actively respond to mRNA vaccines for candidate antigens.

### 3.5. Identification of the Relationship between Immune Subtypes and Immunomodulators

**Figure 7:**
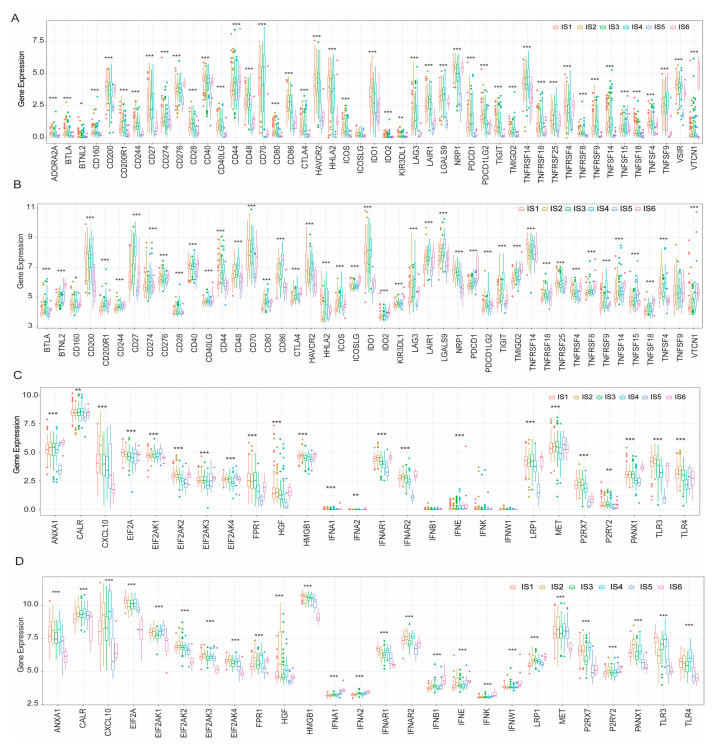
Correlation between tumour immune subtypes and ICP and ICD genes. The expression of ICP genes between different immune subtypes of KIRC in the TCGA (**A**) and GEO (**B**) cohorts. In the TCGA (**C**) and GEO (**D**) cohorts, ICD-regulated genes were differentially expressed in immune subtypes of KIRC. (* *p* < 0.05, ** *p* < 0.01, *** *p* < 0.001). Immune checkpoint (ICP); Immunogenic cell death (ICD); Kidney renal clear cell carcinoma (KIRC); The Cancer Genome Atlas (TCGA); Gene Expression Omnibus (GEO).

Studies have shown that immune checkpoint (ICP) and immunogenic cell death (ICD) modulators play an important role in the regulation of host immunity, thus affecting the efficacy of mRNA vaccines for cancer patients. Therefore, the mRNA differential expression of ICP and ICD modulators was evaluated in KIRC patients with different tumour immune subtypes. In the TCGA cohort, 46 ICP genes (97.9%), except ICOSLG, were differentially expressed between immune subtypes (Figure 7A). Forty-five ICP-related genes were detected in the GEO cohort, of which 44 (except TNFSF9; 97.8%) were differentially expressed between the immune subtypes (Figure 7B). CD276, CD44, and VTCN1 were significantly upregulated in IS6 tumours in the TCGA cohort, whereas BTNL2, CD244, CD276, ICOSLG, TMIGD2, TNFRSF18, KIR3DL1, PDCD1, TNFRSF4, TNFRSF8, TNFSF14, TNFSF18, and VTCN1 were overexpressed in IS6 tumours in the GEO cohort.

In the TCGA cohort, 23 ICD genes (88.5%) were differentially expressed in immune subtypes (Figure 7C). Similarly, 26 ICD genes (100%) in the GEO cohort showed significant differences between immune subtypes (Figure 7D). CXCL10, P2RX7, TLR3, and TLR4 were significantly upregulated in IS2 tumours in the TCGA cohort, whereas ANXA1, CXCL10, FPR1, IFNAR2, P2RX7, and TLR4 showed significantly higher expression levels in IS4 tumours in the GEO cohort. In summary, immunotyping of KIRC tumours reflects the expression levels of ICPs and ICD modulators, which could aid in identifying patients suitable for mRNA vaccination.

### 3.6. Cellular and Molecular Characterisation of Tumour Immune Subtypes

**Figure 8:**
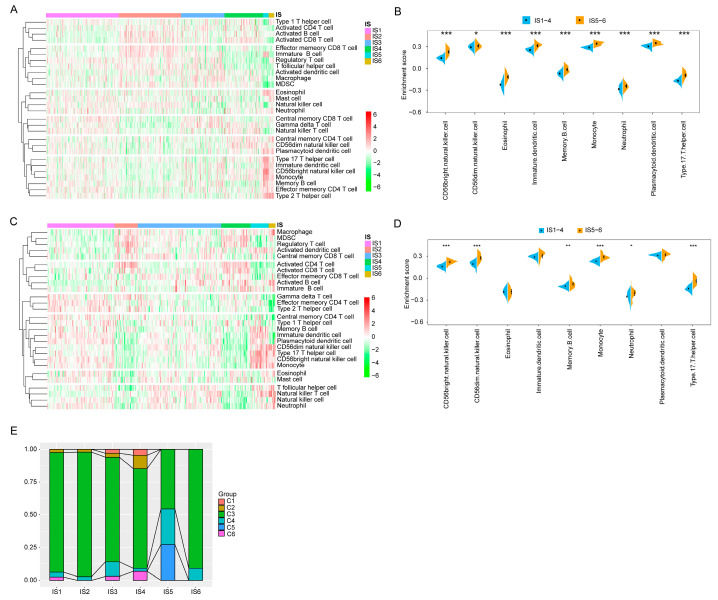
Cellular and molecular characterisation of tumour immune subtypes. In the TCGA (**A**) and GEO (**C**) cohorts, 28 immune cell signatures were differentially enriched in KIRC immune subtypes. Differential enrichment scores of 28 immune cell signatures in the TCGA (**B**) and GEO (**D**) cohort. (**E**) Proportion of overlap between the KIRC immune subtypes and the six pan-cancer immune subtypes. The Cancer Genome Atlas (TCGA); Gene Expression Omnibus (GEO); Kidney renal clear cell carcinoma (KIRC). (* *p* < 0.05, ** *p* < 0.01, *** *p* < 0.001).

The effect of mRNA vaccines in tumour patients depends on the status of TIME. Thus, in TCGA and GEO cohorts, the immune cell components in six immune subtypes were further characterised using ssGSEA. In the TCGA cohort, immune cell components were divided into six clusters, with similar immune cell scores for IS5 and IS6 and similar distribution for IS1–4 (Figure 8A). A marked difference in composition of the infiltrating immune cells was observed between IS1–4 and IS5–6. The scores of CD56bright natural killer cells, CD56dim natural killer cells, eosinophils, immature dendritic cells, memory B cells, monocytes, neutrophils, plasmacytoid dendritic cells, and type 17 T helper cells in IS5 and IS6 were significantly higher than those in IS1–4 (Figure 8B). Thus, IS1–4 is immunologically ‘cold’, that is, characterised by a lack of immune cell infiltration, whereas IS5 and IS6 are immunologically ‘hot’ phenotypes. The results also showed similar trends in the GEO cohort (Figure 8C,D). The mRNA vaccine may induce immune infiltration in patients with immunologically cold IS1–4 tumours. These results suggest that the tumour immune subtypes reflect the individual immune status of KIRC patients and can be further used to determine which patients are suitable for mRNA vaccination.

It has been previously reported that the pan-cancer immune subtypes can be divided into six subtypes (C1–C6), among which KIRC mainly clustered into C3, C4, and C2 [27]. Therefore, the reliability of immune typing in this study can be confirmed by comparing the results of the two types. As shown in Figure 8E, IS1–4 and IS6 mainly overlapped with C3, and IS5 with C3, C4, and C5. Previous studies have suggested that C3 and C6 are associated with better and worse prognoses, respectively, among the pan-cancer immune subtypes, whereas C1 and C2 predict moderate prognoses [29]. These results are in line with the finding that patients with IS5 and IS6 tumours had prolonged survival compared with those with IS3 and IS4. Interestingly, most IS5 patients with a good prognosis and IS4 patients with poor survival overlap with C3. These findings not only prove the reliability of our immunotyping methods but also enrich the previous classification of generalised carcinoma. In conclusion, immune subtypes reflect the immune status of patients with KIRC, and patients with IS1–4 immune cold tumours may be candidates for the mRNA vaccine.

### 3.7. Immune Landscape of KIRC

**Figure 9:**
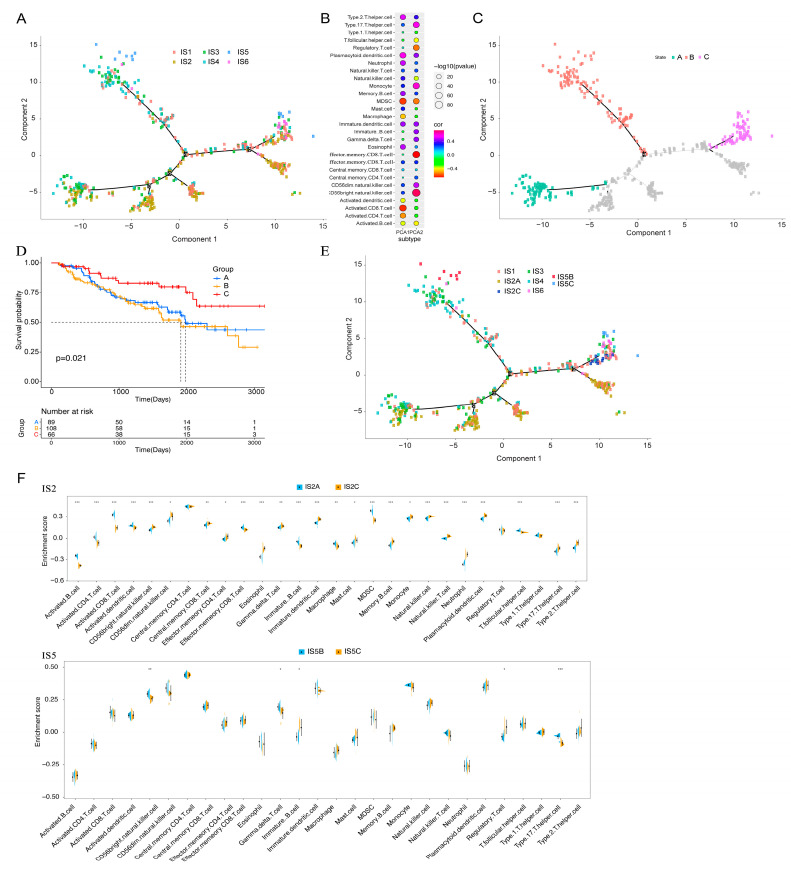
Immune landscape of KIRC. (**A**) Each dot represents a patient, and the immune subtypes are indicated in different colours. (**B**) Correlation between the principal component and immune cell infiltration. (**C**) Patients separated by the immune landscape based on their location. (**D**) Isolated patients have different prognoses. (**E**) Immune landscape of immune subgroups of IS2 and IS5 in KIRC. (**F**) Differential enrichment scores of 28 immune cell characteristics in the IS2 and IS5 subsets. Kidney renal clear cell carcinoma (KIRC); Immune subtype (IS). (* *p* < 0.05, ** *p* < 0.01, *** *p* < 0.001).

The immune landscape of KIRC was constructed using the immune gene expression profiles of individual patients. The horizontal axis is correlated with the abundance of numerous immune cells, among which plasmacytoid dendritic cells, type 2 T helper cells, and memory B cells are most correlated, and the vertical axis is most correlated with effector CD56 bright natural killer cells (Figure 9A,B). The overall immune landscape distribution of IS2 was opposite to that of IS5 and IS6. For IS1, IS3, and IS4 subtypes, the distribution of the same subtype also showed an opposite distribution, indicating significant intra-cluster heterogeneity. After comparing the prognosis of samples with extreme distributional locations in the immune landscape, the survival probability of patients in group C was better than that in groups A and B (Figure 9C,D). According to the distribution location of the immune landscape, IS2 and IS5 were divided into two subsets (Figure 9E), and the enrichment scores of tumour-infiltrating immune cells among the multiple subsets were significantly different (Figure 9F). For example, IS2C showed lower enrichment scores of activated B cells, macrophages, activated CD4+ T cells, activated CD8+ T cells, effector memory CD8+ T cells, and myeloid-derived suppressor cells (MDSCs), whereas IS5C scored lower about CD56bright natural killer cells, gamma delta T cells, and type 17 T helper cells. In summary, the immune landscape based on immune subtypes can accurately identify the immune-related cells and molecule components of each KIRC patient and predict the patient’s prognosis, providing favourable conditions for the personalised selection of mRNA vaccines for tumour treatment.

### 3.8. Identification of Co-Expression Modules and Hub Genes of Immune Genes

**Figure 10:**
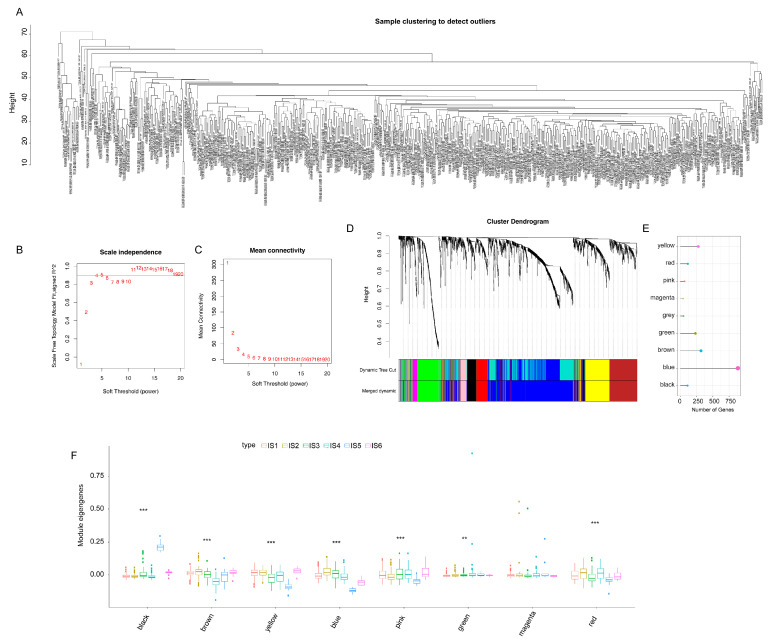
Identification of co-expression modules of immune genes in KIRC. (**A**–**D**) Gene co-expression network analysis based on immune-related genes in KIRC. (**A**) Sample clustering. (**B**) Scale-free fit index. (**C**) Mean connectivity. (**D**) Dendrogram of all differentially expressed genes. (**E**) Gene numbers of co-expressed modules. (**F**) The distribution of the six immune subtypes in the eigengenes of eight modules. Kidney renal clear cell carcinoma (KIRC). (** *p* < 0.01, *** *p* < 0.001).

**Figure 11:**
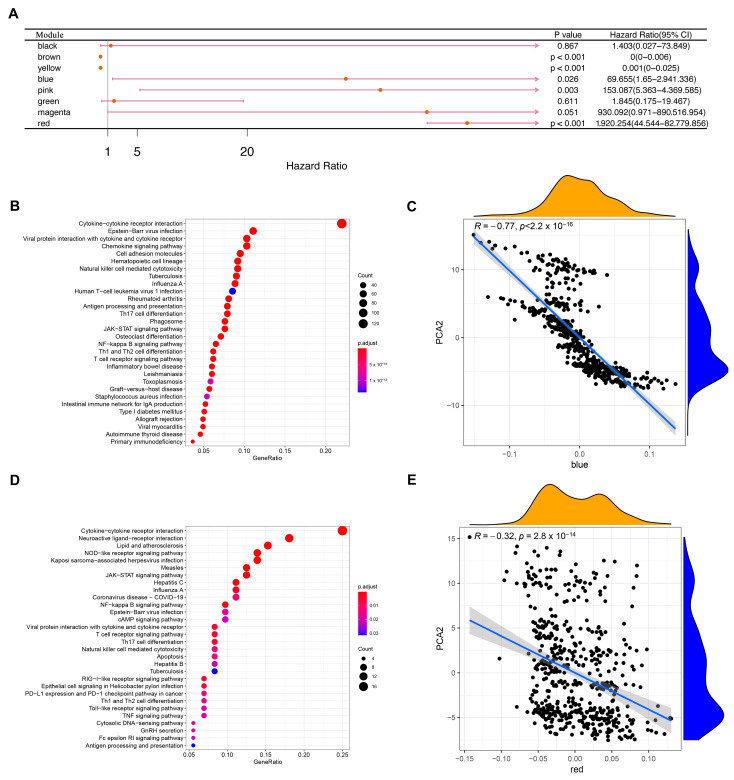
Identification of KIRC immune-related hub genes. (**A**) Single factor survival analysis of blue and red modules in KIRC. (**B**) A dot plot showing the top 30 biological processes in the blue module. (**C**) Correlation between feature vectors of the blue module and the second principal component in the immune landscape. (**D**) The red module shows the dot plot of the top 30 biological processes. (**E**) Correlation between red module feature vectors and the second principal component in the immune landscape. Kidney renal clear cell carcinoma (KIRC).

WGCNA was used to identify immune gene co-expression modules (Figure 10A–C). Nine co-expression modules with 2108 transcripts were obtained (Figure 10D), among which the grey module gene was not clustered with other genes (Figure 10E). After further analysis of the distribution of the six immune subtypes in the eigengenes of eight (except grey) modules, the module eigengenes of IS5 and IS6 were remarkably lower in the blue module (Figure 10F). Further correlation analysis of modules and prognosis showed that brown, yellow, blue, pink, and red modules were significantly correlated with the prognosis of KIRC. In the TCGA cohort, patients with higher eigengene scores in blue (HR = 69.655, *p* = 0.026) and red (HR = 1920.254, *p* < 0.001) modules were associated with a short OS (Figure 11A). Moreover, the blue module enriched with genes in cytokine–cytokine receptor interaction showed a negative correlation with component 2 of the immune landscape (Figure 11B,C). Similarly, the red module—which is associated with cytokine–cytokine receptor interaction, the JAK-STAT signalling pathway, and the TNF signalling pathway—also showed a consistent negative correlation (Figure 11D,E). Therefore, the mRNA vaccine may not be suitable for patients with low expression of immune-related genes clustered in the blue and red modules. Finally, 13 hub genes (the immune-related genes with a correlation > 90% with the module eigengenes) in the blue and red modules were identified, namely SASH3, VAV1, IL10RA, ARHGAP30, CD53, IKZF1, ITGAL, NCKAP1L, LCP2, FERMT3, IL12RB1, CCR5, and IRF9, all of which are potential biomarkers for selecting suitable patients for an mRNA vaccine. The infiltration and activation of immune cells in tumour tissue, as well as their interaction with immunosuppressive cells, mainly determine the ultimate therapeutic effect of the mRNA vaccine on cancer patients with specific immune subtypes.

## 4. Discussion

KIRC tumour has the highest mortality among different types of renal cancer [1]. At present, most patients diagnosed with KIRC are in the advanced stage of the disease, and surgical resection is the main treatment. However, about 30% of patients have a poor prognosis, such as cancer metastasis and death after surgical resection, indicating the limited therapeutic effect of surgery on patients with KIRC [4]. The treatment of RCC has changed from non-specific immune pathways and targeted therapy directed against vascular endothelial growth factor (VEGF) to immune drug therapy [30]. Currently, stimulating the host immune system in immunity therapy to remove tumours is a hotspot in the field of anti-tumour research.

As far as we are aware, this is the first study to screen KIRC antigens on a large scale based on immune profiles for the development of an mRNA vaccine. By constructing the abnormal expression and mutational landscape of KIRC, a series of targeted antigens, including LRP2 and DOCK8, were identified as promising mRNA vaccine candidates for KIRC. Importantly, we collected 19 KIRC samples and verified the high expression of DOCK8 and LRP2 in tumour tissues, confirming the feasibility of their development as mRNA vaccines. Furthermore, the high expression of selected antigens was positively correlated with OS, RFS, and APC and B cell infiltration. Therefore, these antigens play a crucial role in the development and progression of KIRC, and if adequate APC and lymphocyte infiltration are present, these antigens can be directly processed and presented to T cells to induce immune attack. Although these candidate genes must be functionally proven to be effective as vaccines, their potential for mRNA vaccine development has been supported by previous reports. For example, as a receptor, LRP2 plays an important role in the physiological and nervous systems of several organs, including the kidney, lungs, and intestines, and has been implicated in the development of fibrosis-related diseases and breast and prostate cancers [31]. In patients with congenital pulmonary airway malformation, functional destructive mutations of LRP2 were also found to be highly relevant in lung development and cancer [32]. Continuous expression of LRP2 in melanoma is critical for cell maintenance, and low expression of this gene significantly reduces melanoma cell proliferation and survival [33]. Statistical analysis also showed that LRP2 was remarkably associated with a high TMB [34]. DOCK8 is a gene that encodes guanine nucleotide exchange factor and is usually highly expressed in lymphocytes. Mutations in this gene cause DOCK8 deficiency, which is known as autosomal recessive hyperimmunoglobulin E syndrome (AR-HIES). In humans, DOCK8 deficiency leads to combined immunodeficiency disease (CID), which is clinically associated with chronic infection of a variety of microbial pathogens and is conducive to the development of malignant tumours [29,35]. The DOCK8 gene and epigenetic inactivation are involved in the development of lung cancer and other cancers by interfering with cell migration, morphology, adhesion, and growth [36]. It was also shown that DOCK8 protein can regulate macrophage migration [37]. In addition, the high expression of DOCK8 indicates that patients with HPV-positive head and neck squamous cell carcinoma (HNSCC) have a good prognosis and an elevated level of microenvironmental immune infiltration [38]. The evidence suggests that, as an immunostimulatory molecule, DOCK8 can promote immune cell infiltration. Taken together, DOCK8 can stimulate the patient’s immune system and is an ideal antigen for KIRC (especially for patients with DOCK8 mutations via intratumoural injection).

Given that the benefits of survival and therapeutic responses of patients subjected to mRNA vaccine-based cancer immunotherapy remain limited to a small population, KIRC was divided into six immune subtypes according to the immune gene expression profile and different molecular, cellular, and clinical characteristics to select the appropriate population for vaccination. The prognosis of patients with IS5 and IS6 tumours was better than other subtypes in the GEO and TCGA cohort, and patients with IS1–4 tumours associated with higher TMB and somatic mutation rates may be more responsive to mRNA vaccines, suggesting that immunotyping can be used to predict the prognosis of KIRC patients and their response to mRNA vaccines. According to previous immunotyping, KIRC was divided into subtypes C1–C6, with better prognosis for C3, moderate prognosis for C1 and C2, and poor prognosis for C6. [32]. In our study, KIRC was divided into subtypes IS1–6, in which C3 and C6 were associated with better and poor prognosis, respectively, whereas C1 and C2 suggested moderate prognosis. Patients with IS5 and IS6 tumours had prolonged survival compared to those with IS3 and IS4, which is also consistent with previous findings. Interestingly, however, most IS5 patients with a better prognosis and IS4 patients with a poor survival rate overlapped with C3. Therefore, the immunotyping method presented in this paper is reliable and a good supplement to the previous classification methods.

Because tumour immune status is a determinant of the efficacy of the mRNA vaccine, it is necessary to further characterise the immune cell components of different subtypes [39]. IS5 and IS6 showed significantly elevated scores of CD56 bright natural killer cells, CD56 dim natural killer cells, neutrophils, eosinophils, plasmacytoid dendritic cells immature dendritic cells, memory B cells, monocytes, and type 17 T helper cells compared to IS1–4. This indicated that IS5 and IS6 were immunologically hot and that IS1–4 was immunologically cold, which indicates a lack or paucity of immune cell infiltration. To avoid a low level of immune response in IS1–4 tumours, the use of an mRNA vaccine to stimulate the immune system may be an appropriate option. Because the presence of immune checkpoints affects the immune response to tumours, ICBs combined with other tumour therapies can induce ICD in tumours, thereby enhancing the anti-tumour effect [40]. In the GEO and TCGA cohort, the high expression of ICPs in IS6 tumours may inhibit the immune response induced by the mRNA vaccine. However, the expression of ICD modulators in IS1–4 is higher than that in IS5 and IS6, suggesting that the mRNA vaccine may have greater potential in these immune subtypes. These results showed that the molecular signatures of these immune subtypes were consistent with the cellular characteristics. In addition, the complicated immune profile of KIRC indicates considerable heterogeneity between individual patients, rendering personalised mRNA vaccine therapy even more important.

The distribution location of the immune landscape revealed intra-cluster heterogeneity in immune subtypes. For example, in IS2C tumours with immune cold and immunosuppression, low CD8+ T cells and lymphocytes can be combined with ICB or ICD modulators to restore immune system vitality, thereby increasing immune cell infiltration. It is very important to comprehensively consider the patient’s immune landscape and immune subtype to screen the appropriate subpopulation for mRNA vaccination. In blue and red modules, the expression of 13 hub genes, namely SASH3, VAV1, IL10RA, ARHGAP30, CD53, IKZF1, ITGAL, NCKAP1L, LCP2, FERMT3, IL12RB1, CCR5, and IRF9, were negatively correlated with immune component 2; thus, patients with high expression of these genes may respond to mRNA vaccines. This paper has some limitations for which further experimental verification is needed to validate these findings.

## 5. Conclusions

Based on a comprehensive analysis of the immune characteristics and prognosis of KIRC cells, LRP2 and DOCK8 are potential tumour antigens for mRNA vaccine development and are suitable for IS1–4 patients. These results will be helpful for the development of an anti-KIRC mRNA vaccine, prediction of patient prognosis, and selection of appropriate patients for vaccination.

## Data Availability

The data presented in this study are available on request from the corresponding author.

## Supplementary Materials

The following supporting information can be downloaded at: https://www.mdpi.com/article/10.3390/vaccines11020396/s1, Figure S1: Overview of study design; Table S1: Clinical information of samples.

## Abbreviations

APC: antigen-presenting cells; aRCC: advanced renal cell carcinoma; DCs: dendritic cells; DOCK8: dedicator of cytokinesis 8; ICB: immune checkpoint blockage; ICD: immunogenic cell death; ICPs: immune checkpoints; IVT: in vitro transcribed; KIRC: kidney renal clear cell carcinoma; LRP2: LDL receptor related protein 2; MET: MET proto-oncogene survival receptor tyrosine kinase; MDSCs: myeloid-derived suppressor cells; OS: overall survival; RCC: renal cell carcinoma; RFS: relapse-free survival; ssGSEA: single-sample GSEA; TCGA: The Cancer Genome Atlas; TIICs: tumour-infiltrating immune cells; TIME: tumour-immune microenvironment; TMB: tumour mutation burden.
