# Supplementary material for: LRP2 and DOCK8 Are Potential Antigens for mRNA Vaccine Development in Immunologically ‘Cold’ KIRC Tumours
Source: Vaccines (Basel). 2023 Feb 9;11(2):396. doi: 10.3390/vaccines11020396 (PMC9966310; doi:10.3390/vaccines11020396)
Supplement: Supplementary file 1 [file vaccines-11-00396-s001.zip › vaccines-2090994-supplementary.pdf]

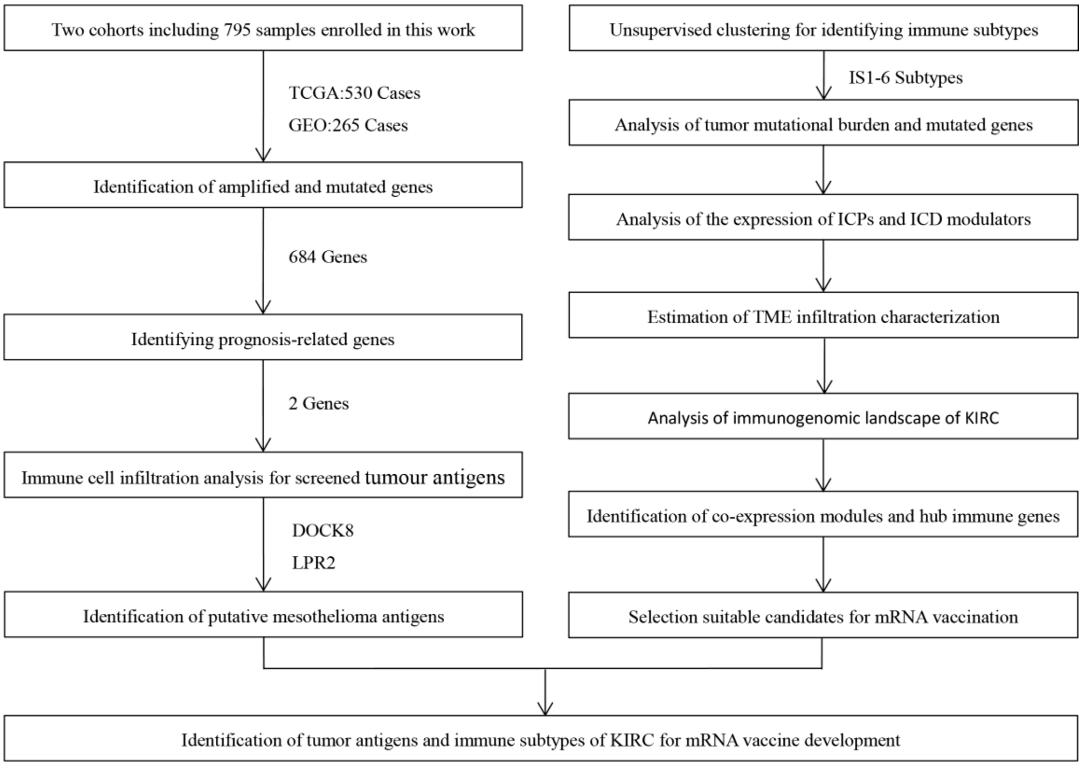

Figure S1. Overview of study design. The Cancer Genome Atlas (TCGA); Gene Expression Omnibus (GEO); Immune subtype (IS); Immune checkpoints (ICPs); Immunogenic cell death (ICD); Tumour microenvironment (TME); Kidney renal clear cell carcinoma (KIRC).

Table S1. Clinical information of samples.

Variable	Number of Samples	%
Gender		
FEMALE	191	35.6
MALE	346	64.5
Age		
≤65	352	65.5
>65	185	34.5
Stage		
I	269	50.1
II	27	10.6
III	125	23.3
IV	83	15.5
Missing	3	0.6
Grade		
G1	13	2.6
G2	230	42.8
G3	207	38.5
G4	78	14.5
Missing	8	1.5
T-stage		
T1	275	51.2
T2	69	12.8
T3	182	33.9
T4	11	2.0
